# Complications of Gonorrhoea in Women

**Published:** 1909-01

**Authors:** F. G. Hodgson

**Affiliations:** Atlanta, Ga.


### COMPLICATIONS OF GONORRHOEA IN WOMEN.

BY F. G. HODGSON, M. D., ATLANTA, GA.

Attention was first called to the importance of this disease by Bernutz and Goupil, as early as 1857, but the real gravity of the affection was not appreciated until after the appearance of Noeggerath's monograph in 1872. The gonoccocus was first described by Neisser in 1879.

Gonorrhoea is usually contracted by sexual intercourse, but it undoubtedly may occur, especially in young girls by indirect infection from soiled clothes, water closets, etc. The gonococci once established in human body, may remain alive for ten to fifteen years or more.

In women the primary infection is usually in the urethra. The squamous epithelium of the vulva and vagina in adults is more resistant than the urethral or cervical epithelium. Children, however, readily develope a vulvo-vaginitis on account of the more delicate epithelium. Next to the urethra the cervical mucosa is most frequently attacked and this is followed by an endometritis.

Bartholins glands are not attacked until later, and this may be followed by inguinal adenitis or "bubo."

The symptoms of this disease usually begin with frequent and painful micturition due to the urethritis and often a cystitis. These symptoms may be very mild and not sufficiently troublesome to cause the woman to consult a physician. The next thing noticed is a purulent discharge which comes from the cervix and later the body of the uterus. This also may be overlooked or mistaken for a simple leucorrhoea. So it is possible for a woman to have gonorrhoea and to be ignorant of the fact. It does not, however, often continue this mild course. After unusual exertion, or excess, or after abortion or pregnancy the gonococci begin to grow very rapidly and finding the parts in a suitable condition they set up serious symptoms.

The germs invade the body of the uterus and produce purulent endometritis, they invade the walls and we have an enlarged and tender uterus. Menstruation becomes very painful, the flow prolonged and profuse. The infections then passes into the fallopian tubes and a salpingitis or phyosalpinx results. An exudate of lymph occurs on the peritoneal surface of the tubes and adhesions from between them and the surrounding structures. The ovaries are next affected by contanuity of by an escape of the purulent discharge from the abdominal ostium of the tubes. Then we get more exudate and more adhesions until the tubes and ovaries become bound together and to the adjacent structures in one mass. By this time we have a well-marked pelvio-peritants. The structures are so bound together that they sometimes look as if cement had been poured into the pelvis and allowed to harden. In other cases a district abscess is formed in the cul de sac of Douglas and the adhesions are not so firm. When once infected these parts are subject to recurring attacks of pelvic peritontis.

In regard to conception, it is estimated that 70 to 90 per cent. of cases of sterility are due to gonorrhoea. Many women once infected are never able to conceive—this is an important factor in the so-called "race suicide." In France where gonorrhoea is so prevalent, the birth rate is very low. If an infected woman does conceive she is more prone to abortion on account of the diseased condition of the endometrium.

Another complication which may arise as a result of the disease or strictured condition of the tubes is *ectopic gestation,* and this is as you know, a serious condition.

Another serious complication which may arise, especially in a pregnant woman is pyelitis. The inflammation extends from the urethra into the bladder and thence up the ureters. The ureters are pressed upon by the enlarged uterus and there is a partial retention of urine in the pelves of the kidneys. This forms a suitable condition for the growth of the gonococci and the development of an inflammation. So we get a pyelitis. The next step in the progress of the disease is a pyelonephritis and then a pus kidney.

We have spoken of a pelvic peritonitis. In some cases this may extend and cause a general peritonitis. In other cases a general peritonitis may develope rapidly when the local pelvic symptoms are not marked and it may be mistaken for a peritonitis of appendical or other septic origin.

We have mentioned latent gonorrhoea with few or no local symptoms. These women may conceive and if none of the above complications arise they may bear fairly healthy children, but it has been shown by Lobenstine and Harrar that these children usually weigh less than children of healthy mothers. The initial loss of weight is greater and they regain their birth weight slower. They are more prone to have elevation of temperature and intestinal disturbances. But these are minor considerations when compared to that greatest of dangers, *opthalmia neonatorum.* A committee has recently been appointed by the American Medical Association to investigate this disease and to try to adopt some method of controlling it. The writer represents the State of Georgia on that committee. You all know what a large percentage (about 60 to 70 per cent.), of the inmates of blind asylums owe their wretched condition to this disease, which is a preventable one, and it is a reflection upon society and especially upon the doctors that so many cases occur.

Now consider for a moment that the gonorrhoeal mother has escaped these numerous complications mentioned, and has born a fairly healthy child, and you have prevented ophthalmia by Crede's method she still is not safe. The gonococci may have remained latent so far but the uterus in the puerperal state forms a favorable medium for the growth and spreading of these germs. About the fifth day post partum or later we find a rise in temperature, pain and tenderness in the pelvis. The germs have ascended and attacked the tubes, and the chance for more children is gone. This has become so prevalent that we have the condition known as "one child sterility."

There is still another and more serious complication of gonorrhoea; besides the local manifestations of the disease the germs may enter the general circulation and produce a true bacteraemia. This may manifest itself as an inflammation in a joint and is known as gonorrhoeal rheumatism; or a true septicaemia may develope and the cocci attacking the heart valves produce a gonorrhoeal endocarditis which is usually fatal. A few cases of pyaemia with metastatic abscesses, phlebitis, pleurisy, and meningitis, all of gonorrhoeal origin, have been reported.

Dr. Alex Doctor says that venereal diseases are more serious than cancer or tuberculosis, for they produce more suffering and cause more deaths. Whether we accept this extreme view or not, it indicates to what an enormous exent these diseases prevail. It has been said by Dr. Tabor Johnson that 70 to 95 per cent. of all abdominal operations are caused by gonorrhoea.

The worst features of the disease are: First, that so many women contract gonorrhoea in a perfectly innocent manner and may be wrecked for life, if not killed, in fulfilling the most sacred duty of life; and secondly that so many innocent babes have their sight destroyed and are doomed to a life of darkness and dependence, due to no fault of their own.

BIBLIOGRAPHY.

1. Noeggerath's Monograph, Brown, 1872.
2. Palmer Findlay, Amer. Med., March 17, 1906.
3. J. Wesley Bovee, Amer. Jour. of Surg., Aug., 1906.
4. Alex Doctor, Zeit. f. Gyn., Dec. 2, 1905.
5. Frs. de Martigny, Jour. de Med. et Chir., Sep. 22, 1906.
6. Guy L. Hunner, Am. Jour. of Obstet., Vol. LIV. p. 337.
7. H. J. Boldt, Ibid, Vol. LV. p. 449.
8. Brooke M. Anspach, Ibid. Vol. LV. p. 467.
9. E. P. Davis, Ibid. Vol. LV. p. 474.
10. Janet, Gaz. de Gyn. Feb. 15, 1907.
11. Lobenstine and Harrar, Bull. Lying In Hosp., Dec., 1906.

